# Retinoids induce cellular senescence in breast cancer cells by RAR-β dependent and independent pathways: Potential clinical implications (Review)

**DOI:** 10.3892/ijo.2015.3013

**Published:** 2015-05-18

**Authors:** ANNE SHILKAITIS, ALBERT GREEN, KONSTANTIN CHRISTOV

**Affiliations:** Division of Surgical Oncology, Department of Surgery, University of Illinois at Chicago, Chicago, IL 60612, USA

**Keywords:** senescence, retinoids, retinoid receptors, breast cancer cells

## Abstract

Most studies on cellular senescence (CS) have been performed *in vitro* by employing cytotoxic agents, irradiation, chromatin and telomerase modulators or by activating certain oncogenes. All these approaches usually lead to DNA damage, gene instability and/or chromatin alterations that primarily affect p53-p21 signaling. Little is known on whether retinoids and rexinoids, which are cell differentiation agents, can also induce CS *in vitro* and *in vivo,* and which molecular mechanisms are involved in promoting the senescent phenotype. We reviewed the recent publications on CS induced by retinoids and rexinoids in ER^+^ and ER^−^ breast cancer cell lines and in corresponding animal models of mammary carcinogenesis which simulate those of human breast cancer. The role of retinoic acid receptors β2 and 5 (RARβ2 and RARβ5) and of receptor independent genes involved in mediating the senescence program of retinoids and rexinoids in ER^+^ and ER^−^ breast cancer cells is discussed. Potential strategists for clinical implication of CS as biomarker of prognosis and of response to treatment with retinoids, rexinoids and with other cell differentiation and antitumor agents are outlined.

## 1. Introduction

The results from a breast cancer prevention clinical trial in the past have shown that 4-hydroxyphenylretinamide (4-HPR, fenretinide), a synthetic retinoid, given for more than 5 years to women with removed primary breast cancer suppressed by 30% the development of second cancer in the contra-lateral breast (1). Most importantly, 4-HPR decreased the incidence of both, ER^+^ and ER^−^ tumors that is not the case with tamoxifen and aromatase inhibitors. 4-HPR was particularly efficacious in premenopausal women, suggesting potential involvement of ER/PR signaling in mediating the antitumor potential of retinoids (2). However, because of some side-effects of 4-HPR, these early clinical studies were not extended and over the last 25 years no further large scale breast cancer prevention trials with retinoids have been performed (3). In addition to 4-HPR, all-*trans* retinoic acid (atRA, tretinoin), 9-*cis* retinoic acid (9-*cis* RA, alitretinoin), 13-*cis* retinoic acid (13-*cis* RA, isotretinoin) and rexinoid, LGD1069 (targretin, bexarotene) have been also used for treatment of breast and other types of cancer, but in most cases disappointing clinical results have been reported (4). Surprisingly, the combination of retinoids with temoxifen (5,6) or with chemotherapy agents (taxol, cisplatin and histone deacethylase inhibitors) did not significantly improve the clinical outcome in patients with metastatic breast cancer (7). Most studies suggest that retinoids suppress cell and tumor growth by receptor dependent and independent mechanisms (3,4). Retinoids are ligands of retinoic acid receptors alpha, beta, gamma (RARs, α, β and γ), whereas rexinoids are ligands of retinoid X receptors alpha, beta, gamma (RXRs, α, β and γ). Both, retinoids and rexinoids affect normal and tumor cells by modulating transcriptional activity of the above receptors, as well as by exploring receptor independent mechanisms (8,9). Retinoids and rexinoids are cell differentiation agents, which induce differentiation of both, epithelial and non-epithelial cells that consequentially leads to inhibition of proliferation (10). Previously, we have shown *in vivo* that retinoids (atRA, 9cRA and 4-HPR), rexinoids (LGD1069), tamoxifen, aromatase inhibitors (vorazole) and DHEA, in addition to inhibition of cell proliferation can also induce CS in premalignant lesions and tumors of MNU-model of mammary carcinogenesis which develops ER^+^ tumors in rats (11,12). For both, retinoids and rexinoids, lower doses preferentially suppressed cell proliferation and induced CS, whereas higher doses induced apoptosis (13). Recently, we found that rexinoids (bexarotene, LGD1069, targretin) are also efficacious inhibitors of mammary carcinogenesis in MMTV-Neu mice, which spontaneously develop ER^−^ mammary tumors similar to those of triple negative Her2/Neu positive breast cancers (14). The antitumor potential of rexinoids in this model was associated with decreased cell proliferation and increased CS. Cytotoxic agents, which cause DNA damage and gene instability can also induce CS by activating p53-p21 signaling (15,16). Each of the above cellular mechanisms is consequence of multiple and well orchestrated gene alterations recently summarized in several excellent reviews (17–19). Over the last several years, intensive research has been done on the role of oncogenes in the development and maintenance of senescence phenotype in normal and tumor cells. Among various oncogenes, the level of MYC and RAS expression appears to play critical role. It was found that they may promote or suppress tumor progression and in the latter CS plays a significant role (20,21). Increasing evidence indicates that SC are metabolically active and may secrete various cytokines, which may not only inhibit, but also promote cell proliferation and eventually tumor progression (18,22,23).

## 2. Retinoids and rexinoids differentially modulate senescence associated genes in ER^+^ and ER^−^ breast cancer cells

**Figure 1:**
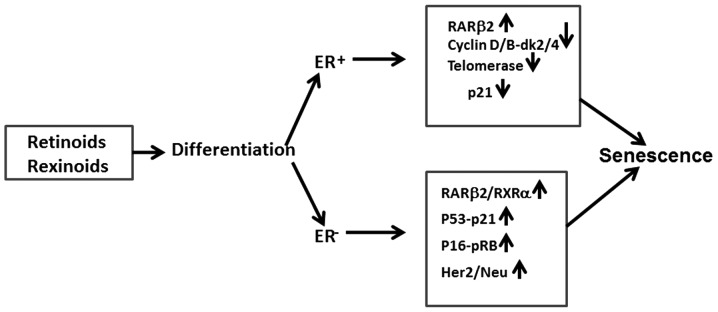
Summarized data on the biological effects of retinoids and rexinoids on mammary tumor cells. At physiological doses retinoids and rexinoids induce differentiation which in ER^+^ cells upregulate RARβ2 expression, but decrease cyclinD/B-cdk2/4, telomerase and p21 expression leading to senescence. In ER^−^ cells retinoids and rexinoids induce RARβ2 and RXRα, p53-p21, p16-pRB and Her2/New expression also leading to senescence. Differentiated cells may also die by non-apoptotic cell death.

Studies from our and other laboratories have shown that in ER^+^ breast cancer cell line retinoids (atRA, 9cRA and 4-HPR) are more efficacious than rexinoids (LGD1069, bexarotene, targretin) in inhibiting cell growth and in inducing CS, whereas rexinoids have very similar effect in both, ER^+^ and ER^−^ cell lines (4,10,14,17). ER^+^ breast cancer cells when cultured for a long time, for instance in colony formation assay, are prone spontaneously to senesce contrary to ER^−^ cells, which rarely senesce, but rather develop stem cell phenotype (24). Further analysis of breast cancer cell types revealed that, luminal A and normal-like luminal cells are those that senesce, contrary to luminal B and basal-like cells, which rarely senesce and behave as stem cells. These data are important because human breast carcinomas could be divided into the above subtypes and, thus, their cellular mechanisms of response to treatment could be predicted. In addition to ER status, p21 expression appears also to modulate the retinoid/rexinoid induced CS in normal human mammary epithelial cells (HMECs) and in most breast cancer cell lines (Table I). p21 induction is usually result of DNA damage that leads to p53 activation and consequently to cell cycle arrest, CS and/or apoptosis (16,19). This is well documented for MCF-7 cells treated with doxorubicin, but little is known whether retinoids and rexinoids may also affect p53 and p21 expression. Gene analysis of MCF-7 cells treated with atRA or doxorubicin revealed overlapping of gene alterations, suggesting that in inducing CS retinoids may explore, at least in part, the signaling pathways of genotoxic agents (25). This was also confirmed in our studies on MDA-MB-231 cells treated for 24 h with bexarotene and doxorubicin, where p21 was upregulated (14). The extension of treatment with bexarotene from 1 to 3 days increased not only p21, but p53 expression as well and this correlated with increased gH2A.X level, an indicator of DNA damage. Since, in MCF-7 cells, bexarotene decreased p21 expression, it appears that ER status may differentially modulate the molecular mechanisms of response of breast cancer cells to rexinoids and retinoids (Fig. 1). In addition to p53-p21 axe, retinoids and rexinoids may explore other signaling pathways in cell growth inhibition and CS. For instance, decreased cyclin D1/E-cdk4/6 expression by ubiquitination and protein degradation may suppress pRb phosphorylation and E2F expression, leading to temporary (quiescence) or permanent (senescence) cell proliferation arrest (26). Similar data were recently reported for cyclin D1^KE/KE^ deficient mouse MECs that express ErbB2 (27). MECs developed autophagy, but failed to implement ErbB2-induced senescence *in vivo*. Downregulation of autophagy or cdk4/6 activity in MECs led to decreased autophagy and increased CS. We also found that bexarotene decreased ATG4B (autophagy related 4B cystein peptidase) in ER^+^, T47D cells but had opposite effect in resistant to senescence ER^−^, MDA-MB-231 cells (Table II). It was also found that p95HER2 expression, a constitutively active fragment of the tyrosine kinase receptor HER2 may result in either increased cell proliferation or senescence (28). In SC, p95HER2 elicits a secretome enriched in proteases, cytokines and growth factors which eventually may increase cell growth and metastatic capacity of breast tumor cells (22). The data support previous studies showing that co-culturing of SC with fibroblasts stimulate proliferation of the latter (18). Thus, depending on the HER2 signaling, CS may play a double role, to suppress or increase tumor growth. This information is important because ~20% of human breast cancers express HerB2/Neu, suggesting potential involvement of CS as biomarker of response in clinical trials with Herceptine and other antitumor agents (Fig. 1). By cDNA microarray hybridization and RT-PCR analysis it was shown that the retinoid-induced (atRA and 4-HPR) growth arrest in MCF-7 cells is associated with strong induction of 13 genes (25). Four of these genes (IGF-binding protein 3, EPLIN, β IG-H3 and FAT10) have anti-proliferative activity that may lead to CS. The function of the induced genes may also account for other cellular effects of retinoids, including proteosome-mediated protein degradation, increased cell adhesion, and retinoic acid synthesis all of them promoting CS (23,26). In normal HMEC, rexinoids (bexarotene) modulate the activity of more than 100 genes (upregulated and downregulated) (29). Sixteen of these genes have been validated by using quantitative RT-PCR and western blotting, among them: RARβ, growth regulatory genes, transcription factors and differentiation markers. Some of these genes are associated with cell cycle arrest, inflammation and CS. It is still an open question whether retinoids/rexinoid, first induce cell differentiation and consequently cell cycle arrest, which when continues for a long time may lead to senescence, or senescence is independent cellular mechanisms not necessarily associated with differentiation, as has been reported for lymphocytic leukemia with remarkable clinical benefits (30). In another study, Wainwright *at al* (31) developed two sub-clones from SK-N-SH neroblastoma cell line: in SH-N sub-clone atRA induced neuronal differentiation with characteristic neurofilaments, whereas in SH-F sub-clone cells senesce with concomitant p16^Ink4a^ and p18^Ink4b^ upregulation and surprisingly with decreased p21 expression. This did not happen with differentiated SH-N sub-clone where atRA induced p21. Thus, it appears that p21 may play distinctive role in mediating cell differentiation and senescence induced by retinoids in various tumor cell types. Cooperation between RARs/RXRs, EGF/TGF/IGF and TGFα, TGFβ1/TGFβ2 signaling has been reported recently (32). In addition to RARs, retinoids may modulate WNT/NOTCH, PI3K/AKT, MAPKs and PKA/PKC signaling and thus reduce cell proliferation and eventually induce CS (33). In a recent study from our laboratory, T47D and MDA-MB-231 cells were treated for 24 h with bexarotene, and gene alterations, some of them associated with CS were identified (Table II). Since, it takes 5–7 days for both retinoids and rexinoids to induce CS *in vitro*, the selected in Table II genes do not directly represent those expressed in SC. However, they do indicate that even at very early time-points, rexinoids may modulate the activity of certain genes that contribute to CS. For instance, bexarotene induced DHRS3 and RARRES3 genes, which are associated with retinoid metabolism and storage and thus by collateral mechanisms may affect RARs and RXRs, including RARβ and consequently CS (34,35). It appears that bexarotene is more efficacious inducer of differentiation in ER^+^, T47D cells than in ER^−^, MDA-MB-231 cells, as demonstrated by upregulation of GDF15, KRT13 and CEND1, genes associated with cell differentiation. In both cell lines bexarotene suppressed cell cycle progression (telomerase reversed transcriptase-TERT, CEND1 and CDK11B), intercellular matrix protein stromolysin 3 (MMP11) and basal membrane (laminin alpha 3-LAMA3) proteins, which indirectly or by paracrine mechanisms potentiate CS (36). Modulation of RAS oncogene (RAB26) and IGFBP6 may also contribute to the LGD1069 induced CS in breast cancer cells (37,38).

## 3. RARβ isoforms and cellular senescence in breast cancer cells

**Figure 2:**
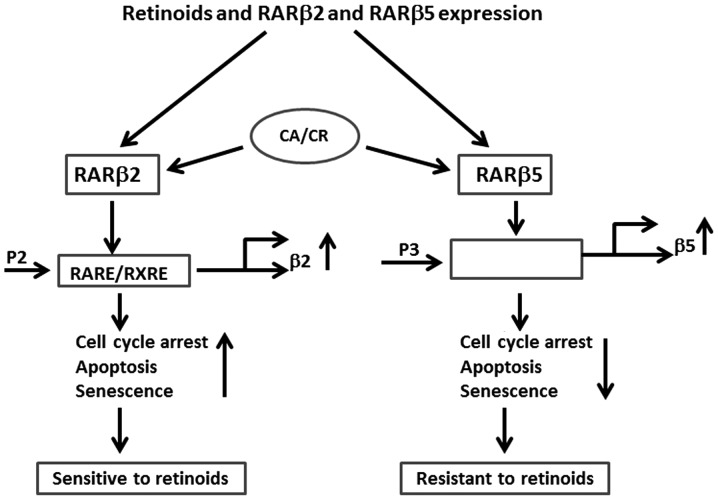
Effects of retinoids on RARβ2 and RARβ5 expression and senescence. Retinoids induce RARβ2 by activating P2 promoter that has retinoic acid response elements (RARE) and retinoid X receptors response elements (RXRE). The upregulation of RARβ2 induces cell cycle arrest, apoptosis and senescence. Retinoids may also induce RARβ5 isoform, which does not have RARE and RXRE in the promoter (P3) and this is associated with stimulation of cell proliferation, and protection of cells to senesce or die by apoptosis. The biological activity of both isoforms could be modulated by co-activators (CA) or co-repressors (CR).

RARβ has five isoforms: β1, β2, β3, β4 and β5 (8,9). RARβ2 and RARβ4 isoforms are mostly examined in breast normal and tumor cells, but they may also mediate the effect of retinoids in other epithelial cell types (8,10). RARβ2 is expressed in normal MECs, but is lost in most breast cancer cells and in most premalignant lesions and tumors, suggesting its tumor suppressor role (39). Activation of RARβ2 by retinoids or by epigenetic approaches, as well as by gene transduction to cells lacking the receptor may lead to decreased proliferation and increased senescence (40,41). Previously, we have identified a novel RARβ (β5) isoform (GenBank: AC133141.2 and AC098477.2) which has an independent P3 promoter and appears to play a dominant negative role in RARβ signaling (42). Breast cancer cells that express RARβ5 were resistant to retinoids and when treated with atRA did not senesce. RARβ5 inhibition by siRNA in MDA-MB-231 and BCA2 cells increased their sensitivity to retinoids, as determined by cell growth inhibition and CS (43). As shown in the Fig. 2, retinoids/rexinoids induce cell cycle arrest and CS by activating P2 promoter and RARβ2 transcription, whereas upregulation of P3 promoter and RARβ5 expression have opposite effect and suppresses CS. At mRNA level, the high RARβ2/RARβ5 ratio was associated with increased cell sensitivity to retinoids (atRA, 9cRA), further supporting the role of RARβ5 as potential dominant negative regulator of RARβ2 (Fig. 2). However, there are breast cancer cell lines, which do not express RARβ5, but are also resistant to retinoids, suggesting involvement of other transcription factors in mediating the cellular effect of retinoids (19,36,38). Recently, it was shown that breast carcinomas with high RARα/RARγ ratio are more sensitive to atRA and have better prognosis than those with inversed ratio of the above receptors (44). By microarray analysis it was found that both, RARs agonists and antagonists produced similar effects on gene expression, suggesting that the RARE-dependent RARβ2 gene transcription is only a partial component of the retinoid-induced cell growth inhibition and CS (45). The ability of retinoids and rexinoids to induce CS depends also on the cell type and genetic background. Thus, in a recent study it was shown that antisense oligonucleotides against RARβ2 reduced proliferation and caused apoptosis in 3 lung cancer cell lines, but had no effect in 2 other cell lines lacking RARβ2, suggesting that RARβ2 may not only suppress, but also promote proliferative activity of tumor cells and thus plays a role of proto-oncogene (41). RARβ isoforms may directly or indirectly cooperate with other RARs and RXRs, ER, and other nuclear receptors (PPARβ/γ, vitamin D, thyroid) and thus affect cellular responses to retinoids and rexinoids (9,41). In most breast carcinomas RARβ is downregulated by hypermethylation of its promoter and/or by alterations of chromatin structure (39,46). Therefore, a combination of retinoids with dimethylating agents, methyltransferase inhibitors or histone deacethylase inhibitors have shown promising efficacy in cell and tumor growth inhibition.

## 4. Retinoids and rexinoids induce CS in mammary premalignant lesions and tumors

**Figure 3:**
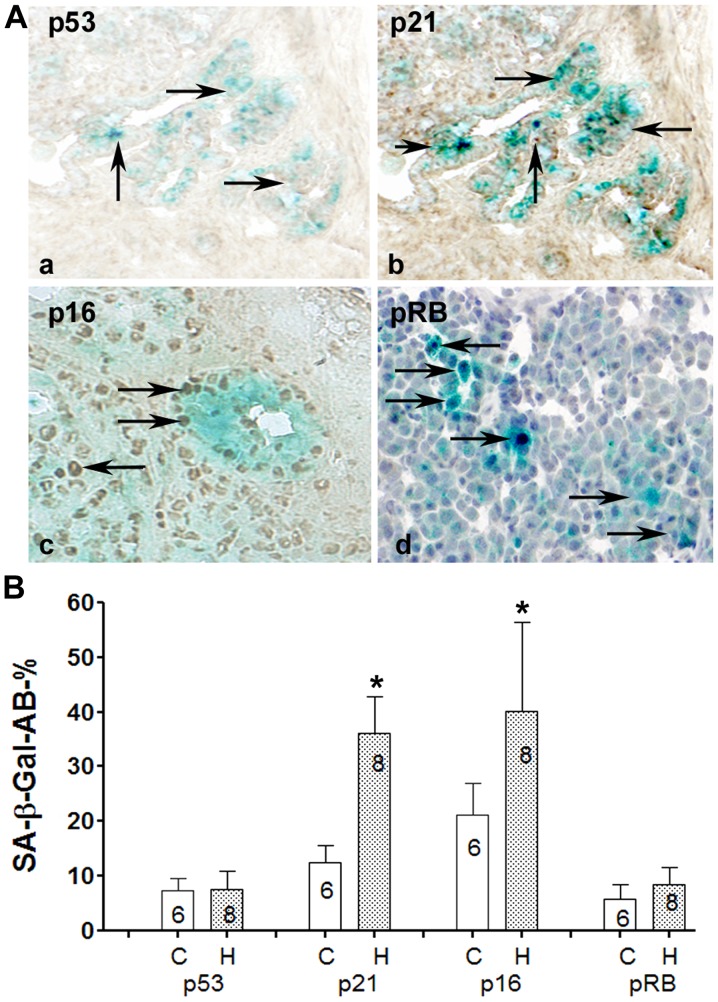
(A) A double-labeling method was developed for identification of genes overexpressed in SC (14). (B) Values of SA-β-Gal cells and the expression of p53, p21, p16 and pRB proteins in senescent and non-senescent cells. C, control values, H, values in treated with bexarotene tumors. In the columns is given the number of animals with tumors examined. (A-a) Frozen sections from mammary tumor of MMTV-Neu mice treated for 4 weeks with bexarotene. Slides were first stained by SA-β-Gal kit and then overnight with antibody against p53. SC (blue stained) are detected among differentiated (alveolar) tumor area. No positive p53 staining in SC as compared to control animals is presented (B, first columns; magnification, ×200). (A–b) Parallel sections from the same tumor stained by SA-β-Gal and p21 antibody, note overexpression of p21 (brown-stained nuclei) among SC also as shown in B (second columns) as well, asterisk indicates significant difference with control values (P<0.05). (A–c) Frozen tumor section from a mammary tumor of animal treated with bexarotene for 4 weeks and double stained by SA-β-Gal and p16 antibody. p16 appears overexpressed (brown-stained nuclei) in SC, as confirmed also in B (third columns; ^*^P<0.05). (A–d) Double staining of frozen tumor section with SA-β-Gal kit and pRB antibody (magnification, ×400). Although, there is increase in pRB in some tumor cells, no significant difference with pRB values in non-senescent cells was found (B, last column).

To identify SC *in vitro* and *in vivo* β-galactosidase (SA-β-Gal) reaction was employed (47). The protocol for conducting this reaction in tissues and tumors and potential alternative methods for identification of SC are described in our previous studies (11–14). We showed that retinoids (9cRA and 4-HPR) at doses that suppress MNU-induced mammary carcinogenesis in rats in addition to inhibition of cell proliferation can also induce CS (11,12). Surprisingly, 4-HPR given for 4 weeks also suppressed telomerase activity that correlated with decreased cell proliferation and increased CS, suggesting the potential involvement of telomerase in the retinoid-induced CS (48). It has been shown that shortening of telomere and decreased telomerase activity lead to gene instability, activate p53 expression, and thus promote CS in p53-dependent manner (49). By employing MMTV-Neu mice, which spontaneously develop ER^−^ mammary tumors, bexarotene given for 4 weeks at 80 or 40 mg/kg body weight, suppressed tumor frequency and growth and this was associated with inhibition of cell proliferation and induction of CS (14). Bexarotene was more efficacious in inducing CS in normal MEC and premalignant lesions than in tumors. SC were predominantly identified in differentiated tumor areas, suggesting that differentiated breast carcinomas are more prone to develop CS than non-differentiated ones (Fig. 3). By double labeling, first with SA-β-Gal to identify SC and then by antibodies that recognize biomarkers expressed in SC, we found that LGD1069 induced RARβ2, p21, p16 and pRB, but not p53 expression in MMTV-Neu mammary tumors, suggesting a p53 independent mechanisms of CS (Fig. 3). To further understand the role of RARβ expression on the retinoid-induced CS *in vivo*, RARβ wild-type (+/+) and RARβ deficient (−/−) mice were employed. We obtained these mice from the laboratory of Pierre Chambon in Strasburg, France. Mice were cross-bread in our laboratory and RARβ expression was determined by DNA analysis. Mammary gland architecture of RARβ homozygous (−/−) mice did not differ from that of wild-type RARβ mice (+/+). Mice of both genotypes were followed up for more than 12 months and no mammary tumors were identified, suggesting that RARβ deficiency alone does not promote mammary carcinogenesis. Mice of both genotypes were treated with 9cRA at 80 mg/kg for 4 weeks and cell proliferation and apoptosis were determined. No difference in the values of BrdU-labeled and SC was found in mammary terminal end buds (TEBs) and lobules of both genotypes, suggesting that RARβ deficiency alone in normal MECs is not the critical target of retinoids in inhibiting cell proliferation and in inducing CS. Thus, it appears that other transcription factors may contribute to the retinoid-induced CS in normal and tumor MECs.

## 5. Potential clinical implications

### Cellular senescence as biomarker of prognosis

Most studies suggest that malignant transformation is associated with lost potential of cells to senesce (18,19). We confirmed this *in vivo,* in mammary premalignant lesions (AH, MIN and CIS) and tumors of rats and mice (12–14). Studies by Collado *et al* (50) on a mouse model of lung carcinogenesis that expresses K-ras12 V12 oncogene showed SC in benign lesions (adenomas), but not in carcinomas, thus supporting our *in vivo* data on mammary carcinogenesis. Clinical data from patients with benign skin lesions (nevi) (51) and human breast cancer (52), further support the role of CS as suppressor mechanism in tumor development and progression. In addition to CS, low proliferating activity and high apoptosis have been also considered biomarkers of good prognosis (13,53). Thus, based on the values of proliferating cells, CS and apoptosis mammary premalignant lesions and tumors could be divided into two categories; one with high percentage of proliferating cells and low percentage of SC and apoptosis, suggesting progression and poor prognosis and another one, with small number of proliferating cell and high number of CS and apoptosis, suggesting regression, disintegration and favorable prognosis. Based on the percentage of SC in premalignant lesions and tumors, Collado and Serrano (54) suggested the implementation of senescence index (SI), as biomarker of prognosis and treatment efficacy, similarly to cell proliferation and apoptosis indexes. Since, ARF-p53-p21 and/or p16-pRB are involved in mediating the senescent program of antitumor agents including retinoids, the lack or decreased expression of the above genes in breast premalignant lesions and tumors may also have a negative effect on spontaneous CS and thus promote carcinogenesis and tumor progression (55). Recent *in vitro* studies on cell lines transfected with HER2/New support our *in vivo* studies on MMTV-Neu mice indicating that activation of this oncogene can promote CS (56). Since, SC can produce cytokines that not only suppress, but also promote cell proliferation, it has been speculated that CS can play a double role, to suppress or promote tumor development and progression (18,22).

### Cellular senescence as biomarker of efficacy

Efficacy studies with established and novel cancer prevention and therapy agents are another avenue for potential clinical implication of CS. By employing MNU-, and MMTV-Neu models of mammary carcinogenesis, we found that retinoids (9cRA and 4-HPR), rexinoids (bexarotene), tamoxifen and aromatase inhibitors (vorazole), in addition to inhibition of cell proliferation also induced CS and this correlated with their efficacy to suppress tumor growth (12,14). The role of CS as a biomarker of response was also confirmed in a clinical trial with antitumor agents. Patients with p53 wild-type and mutated-types of breast carcinomas have been treated with neo-adjuvant chemotherapy, a combination of cyclophosphamide, adriamycin and 5-fluorouracil (CAF). In tumors removed 12–87 days after chemotherapy, SC have been identified in p53 wild-type tumors only, suggesting p53 involvement in mediating CS. In addition to p53, p21 and p16 expression appears also involved in mediating CS induced by cytotoxic agents (52). After termination of treatment with antitumor agents, SC remain detectable in tissues and tumors for a long time (weeks, months), as compared to cell proliferation and apoptosis, which are short-term cellular events, therefore, CS may have advantage as biomarkers of response in long-term cancer prevention and therapy studies. However, this need to be confirmed in future large scale cancer prevention and therapy studies.

### Development of novel agents that preferentially induce CS

Development of novel agents that selectively induce CS is another avenue that could be explored in cancer prevention and treatment. These agents apparently need to modulate the activity of genes involved in initiation and maintenance of senescent phenotype. As was shown above, modulation of ER, Her2/New, RAS, RARβ2 signaling may affect the decision of cells to stop proliferating, senesce or die by apoptosis. Since, SC produce *in vitro* cytokines that suppress cell growth by inducing CS it has been suggested also to be employed for selective cytokines *in vivo* experiments (55,56). Some cytokines can also stimulate cell growth, therefore, selection need to be strongly monitored.

## Figures and Tables

**Table I:** Effects of atRA and LGD1069 on cellular senescence in breast cancer cell lines.

Cell line	Type	ER/PR	p21	atRA-SC-%, 1.0 μM	LGD1069-SC-%, 1.0 μM
HMEC	Normal	−	+	38	23
MCF10	Benign	−	+	22	20
MCF10AT	AH	−	+	30	15
MCFCA1a	Tumor	−	−	15	6
				26.2±9.9	16±7.4
MCF-7	Tumor	+	+	65	12
T47D	Tumor	+	+	52	24
BT474	Tumor	+	+	28	15
ZR-75-1	Tumor	+	+	33	18
				44.5±17.1^a^–^c^	17.2±5.1^c^
MDA-MB-468	Tumor	−	−	17	8
MDA-MB-231	Tumor	−	+	10	13
MDA-MB-453	Tumor	−	+	15	16
BT-20	Tumor	−	+	27	20
SK-BR-3	Tumor	−	+	21	11
				18±6.4^b^	13.6±4.6
BCA-1	Tumor	−	+	22	12
BCA-2	Tumor	−	−	3	4
BCA-3	Tumor	−	+	26	15
BCA-7	Tumor	−	+	37	20
				22.0±14.1^b^	12.7±6.7

HMEC, human breast epithelial cells, 6–9 *in vitro* passages; MCF10A cell line, immortal, but benign breast epithelial cell line; MCF10AT cell line was generated by stable transfection of MCF10A cells with Ha-Ras oncogene. When transplanted in nude mice MCF10AT cells develop lesions with characteristics of atypical hyperplasia and carcinoma *in situ* of human breast (see also, ref. 53). MCFCA1a is a malignant breast cancer cell line developed after multiple consecutive transplantations of MCF10AT cells in nude mice. BCA1, 2, 3, 7 cells are early *in vitro* passages of breast cancer cells (passage 4–12) which in biology appear to be closer to primary tumors than to established breast cancer cell lines (ref. 72?).

a Significant difference (P<0.02) in the percentage (%) of SC between ER^+^ and ER^−^ cell lines treated with 1.0 μM atRA;

b between ER^+^ and BCA cells which are ER^−^ (P<0.02), and

c between cells treated with atRA and LGD1069 ER^+^ (P<0.02).

**Table II:** Rexinoids differentially affect senescence associated genes in T47D and MDA-MB-231 cells.

Gene symbol	T47D Bex/Con	MB231 Bex/Con	Gene name
DHRS3	9.84	6.48	Dehydrogenase/reductase (SDR family) member 3
RARRES3	5.61	2.08	Retinoic acid receptor responder 3
GDF15	3.08	2.41	Growth differentiation factor 15
TERT	0.49	0.26	Telomerase reverse transcriptase
CDK11B	0.47	0.24	Cyclin-dependent kinase 11B
MMP11	0.44	0.16	Matrix metallopeptidase 11 (stromelysin 3)
LAMA3	0.32	0.48	Laminin, α 3
KRT13	2.78	0.41	Keratin 13
UBE2E2	0.49	2.03	Ubiquitin-conjugating enzyme E2E2
ATG4B	0.48	2.47	Autophagy related 4B, cysteine peptidase
APOD	3.04	0.46	Apolipoprotein D
CEND1	5.05	0.45	Cell cycle exit and neuronal differentiation 1
RAB26	3.49	0.98	RAB26, member RAS oncogene family
IGFBP6	1.99	2.00	Insulin-like growth factor binding protein 6
RAB40AL	1.03	0.41	RAB40A, member RAS oncogene family-like
p53	1.08	0.91	Tumor protein p53

Gene analysis was performed on ER^+^, T47D and ER^−^, MDA-MB-231 breast cancer cells treated for 24 h with 1.0 μM LGD1069. Genes mostly associated with CS are presented. LGD1069 induced. The first three genes: DHRS3, RARRES3 and GDF15, which are mostly associated with cell differentiation and retinoid metabolism are induced in both cell lines. The second group of genes that reflect: CDK11B-cell cycle progression, MMP11-intercellular matrix proteins (stromolysin 3) and LAMA3-basal membrane (laminin α 3) are inhibited in both cell lines. LGD1069 differentially affected: KRT13-keratin 13, UBE2E2-ubiquitin-conjugating enzyme E2E2, ATG4B autophagy related 4B cysteine peptidase, APO-apolipoprotein D, CEND1-cell cycle exit and neuronal differentiation 1, RAB26 and RAB40A, both members of RAS oncogene family, but had no effect on p53 expression suggesting lack of DNA damaging effect. Bex, bexarotene; Con, control.

## Abbreviations

CS: cellular senescence
SC: senescent cells
MNU: N-methylnitrosourea
4HPR: 4-hydroxyphenylretinamide
RARs: retinoid acid receptors α, β, γ
RXRs: retinoid X receptors α, β, γ
